# Albumin Leakage Level during Cytoreductive Surgery and Hyperthermic Intraperitoneal Chemotherapy Is Associated with Major Complications

**DOI:** 10.3390/cancers16162874

**Published:** 2024-08-19

**Authors:** Hyun-Chang Kim, Dong Woo Han, Eun Jung Park, Yeon Hwa Hong, Young Song

**Affiliations:** 1 Department of Anesthesiology and Pain Medicine, Anesthesia and Pain Research Institute, Yonsei University College of Medicine, 50-1 Yonsei-ro, Seodaemun-gu, Seoul 03722, Republic of Korea; onidori@yuhs.ac (H.-C.K.); hanesth@yuhs.ac (D.W.H.); hyh37222@yuhs.ac (Y.H.H.); 2 Division of Colon and Rectal Surgery, Department of Surgery, Gangnam Severance Hospital, Yonsei University College of Medicine, 50-1 Yonsei-ro, Seodaemun-gu, Seoul 03722, Republic of Korea; camp79@yuhs.ac

**Keywords:** albumin, cytoreductive surgery, hyperthermic intraperitoneal chemotherapy, complication

## Abstract

**Simple Summary:**

Many patients undergoing major abdominal surgery experience changes in their albumin levels, but the impact of this on their recovery is not well understood. Our research focused on patients who had cytoreductive surgery and a special chemotherapy treatment during surgery. We looked at how albumin levels changed from the time of surgery until three days after surgery and whether these changes were linked to major complications. We found that patients whose albumin levels dropped significantly during surgery were more likely to have serious complications. By tracking albumin levels, we might better predict and manage complications after this type of surgery. Our findings suggest that monitoring albumin could improve patient outcomes in the future.

**Abstract:**

The clinical consequences of perioperative albumin extravasation accompanying major abdominal surgery remain underexplored. We retrospectively reviewed the data of patients who underwent cytoreductive surgery (CRS) and hyperthermic intraoperative peritoneal chemotherapy (HIPEC). Parameters of albumin kinetics, including serum albumin concentration decrease (∆Alb) and extravasated albumin level (Alb_shift_), were assessed from surgery until postoperative day (POD) 3. Logistic regression analysis identified factors associated with major complications. The association of albumin kinetics with major complications was evaluated using receiver operating characteristic (ROC) curve analysis. Serum albumin levels decreased during surgery and subsequently increased. Of the 121 analyzed patients, 25 (21%) developed major complications. The ∆Alb and Alb_shift_ during surgery and on POD 3 were greater in patients who developed major complications than in those who did not (12 ± 12 vs. 6 ± 14, *p* = 0.032, and 127.5 (71.9) vs. 48.5 (44.9), *p* < 0.001, respectively). Perioperative ∆Alb and Alb_shift_ were associated with major complications. The areas under the ROC curve of Alb_shift_ during the 3 days post-surgery and Alb_shift_ on POD 3 were 0.843 and 0.910, respectively. Alb_shift_ during the 3 days post-surgery and Alb_shift_ on POD 3 were correlated with complications (*p* < 0.05). In conclusion, perioperative albumin loss was associated with major complications in patients undergoing CRS and HIPEC. Alb_shift_ was associated with serious complications.

## 1. Introduction

Albumin is a globular protein that plays a crucial role in sustaining plasma pressure and ensuring balanced nutrition [1]. During major abdominal surgery, plasma albumin levels typically decrease due to surgical trauma-induced inflammation and increased capillary permeability [2,3,4]. Two previous investigations have quantified the decrease in serum albumin (SAlb) concentration (∆Alb) and the extravasation of albumin (Alb_shift_) using SAlb, hematocrit, and hemoglobin levels [3,4]. ∆Alb occurs during the initial part of the surgery and persists until the end of surgery. Alb_shift_ occurs from the start of the surgical procedure until 1 h post-surgery in patients undergoing pancreatic or esophageal surgery [3]; in patients undergoing liver transplantation, Alb_shift_ continues until postoperative day (POD) 3 [2]. Therefore, ∆Alb and Alb_shift_ may be as significant as SAlb levels in assessing the impact of major surgery.

A perioperative decrease in albumin level has been linked with adverse postoperative outcomes in various surgical procedures [5,6,7] and is a predictive factor for pulmonary complications in patients with lung cancer [5]. Hypoalbuminemia has been associated with increased postoperative morbidities and mortalities in lower extremity orthopedic surgeries [7]. Furthermore, diminished postoperative albumin levels are predictive for complications in patients undergoing gastrointestinal surgeries [6]. Despite these findings, the specific impact of ∆Alb and Alb_shift_ on postoperative complications remains unexplored in patients undergoing major abdominal surgery. Cytoreductive surgery (CRS) followed by hyperthermic intraperitoneal chemotherapy (HIPEC) is a viable treatment option for patients with peritoneal metastasis [8,9,10]. It is an intricate and extensive procedure, potentially resulting in severe intestinal trauma, a decrease in albumin levels, and an increase in Alb_shift_. Low preoperative albumin levels are associated with compromised outcomes and reduced survival rates in patients undergoing CRS followed by HIPEC [11,12]. The intraoperative and postoperative changes in SAlb levels, including ∆Alb and Alb_shift_, and their impact on morbidity in patients undergoing CRS followed by HIPEC have not been explored.

In this study, we aimed to comprehensively characterize albumin kinetics in major abdominal surgeries and investigate the prognostic significance of perioperative albumin loss in the occurrence of major postoperative complications.

## 2. Materials and Methods

This retrospective investigation enrolled adult patients aged > 20 years with an American Society of Anesthesiologists’ Physical Status Class 1–3 who underwent CRS and HIPEC at Gangnam Severance Hospital, Seoul, Republic of Korea, between April 2017 and June 2021. The study was approved by the Institutional Review Board of Gangnam Severance Hospital (3-2023-0402) and conducted according to the Strengthening the Reporting of Observational Studies in Epidemiology guidelines [13]. The requirement for obtaining patient consent was waived.

The electronic medical records of patients were meticulously reviewed by three board-certified anesthesiologists (H.-C.K., S.Y.H., and Y.S.) and one resident anesthesiologist (Y.H.H.). The exclusion criteria were a change in the surgical plan after surgical exploration (shifting to CRS only, HIPEC only, or undergoing open and closure procedures) and failure to adhere to the standard protocol for routine blood tests. We excluded data from patients with preoperative infections, particularly COVID-19, to avoid any confounding effects related to the infection. Data from patients who were administered albumin in the intensive care unit (ICU) were not excluded from our study.

All patients underwent CRS followed by HIPEC (CRS + HIPEC) under general anesthesia. All anesthetic and surgical procedures were performed strictly according to our institutional guidelines, as detailed in previous studies [14,15]. Briefly, midline laparotomy was performed, followed by removal of the peritoneum and resection of the metastatic organs to eliminate all visible cancerous tissue. HIPEC involved the infusion of 3 L of hypertonic solution (Dianeal, 1.5% Dextrose Peritoneal Dialysis Solution; Baxter Healthcare Corp., Deerfield, IL, USA) maintained at 42–43 °C. This solution was mixed with three separate doses of mitomycin-C (a total of 35 mg/m^2^) and circulated at a rate of 800–1000 mL/min for 90 min using the Belmont Hyperthermia Pump (Belmont Instrument Corp., Billerica, MA, USA). Subsequently, the resected intestines were anastomosed, and the abdomen was closed. The surgeons opted to create a diversion stoma at their discretion. Hemodynamic parameters such as the cardiac index and stroke volume variation were continuously monitored using the Vigileo^TM^ System (Edwards Lifesciences, Irvine, CA, USA). Central venous pressure was assessed through the internal jugular vein. Anesthesia was maintained using sevoflurane inhalation and remifentanil and rocuronium infusions. Mean arterial pressure was maintained between 60 and 80 mmHg through appropriate fluid resuscitation and norepinephrine administration. Guided by the stroke volume variance and considering preoperative deficit, maintenance, and surgical loss, goal-directed fluid therapy was administered. Up to 1000 mL of primary resuscitation fluid, comprising balanced crystalloids (Plasma Solution-A Inj.; HK Inno. N Corp., Seoul, Republic of Korea) and balanced synthetic colloids (Volulyte; Fresenius Kabi, Bad Homburg, Germany), was administered to compensate for blood loss. Packed red blood cells were transfused if the hematocrit level was <25%. A 20% human albumin solution (SK Chemical, Seoul, Republic of Korea) was used to maintain the SAlb concentration above 3.0 g/dL. Active cooling methods, including the use of a cooling mattress, forced air, and cold fluid infusion, were employed during HIPEC. After surgery, all patients were transferred to the ICU with or without extubation at the discretion of the attending anesthesiologist. In the ICU, colloids were not administered in adherence to our institutional standards. Postoperative care was provided by the attending physician and intensivist. All surgical interventions were performed via open laparotomy, and the duration of HIPEC was 90 min.

### 2.1. Albumin-Related Variables

SAlb, serum hematocrit (SHct), and serum hemoglobin (SHb) were evaluated at the following time points: before surgery; immediately before HIPEC (after CRS); immediately after HIPEC; at the end of surgery; 1 h after surgery; and on PODs 1, 2, and 3.

∆Alb level was defined as the percentage decrease in SAlb level compared with that before surgery. ∆Alb was calculated at the following time points: during CRS, during HIPEC, after HIPEC, during CRS + HIPEC, during 1 h after CRS + HIPEC, during 3 days after CRS + HIPEC, and up to POD 3.

Alb_shift_ was calculated at the following time points: during CRS + HIPEC and up to POD 3. Alb_shift_ was defined as the time-dependent pattern of changes in SAlb levels and albumin extravasation, based on calculations from previous investigations [3,4].

Baseline blood volume (BV) was computed using anthropometry according to body size and sex [16]. Under the assumptions that larger individuals (both in terms of height and weight) generally have a greater blood volume, and that males and females have different body compositions (such as muscle mass and fat distribution), blood volume was calculated using Nadler’s formula. These assumptions allow for the use of anthropometric measurements to predict blood volume accurately for each sex. Plasma volume (PV) was calculated as follows:

(1) $$PV=\left(1-SHct\times 0.91\right)\times BV$$

The calculation of plasma volume (PV) is based on two key assumptions. The formula assumes that the distribution of red blood cells and plasma is uniform throughout the blood volume, except for the correction factor applied. Additionally, the use of a constant correction factor (0.91) assumes that the relationship between the systemic hematocrit and true red blood cell volume fraction is consistent across different individuals. The value of 0.91 of the f-ratio represents the ratio between the total body hematocrit and large-vessel SHct. The intravascular hemoglobin mass (HbM) was calculated as follows:

(2) HbM=BV×SHb

By combining (1) and (2),

(3) PV=(1−SHct×0.91)×HbM/SHb

Intravascular albumin mass (IVAM) can be calculated as follows:

(4) IVAM=PV×SAlb

The formulas are derived under the assumptions that albumin is uniformly distributed throughout the PV and that the SAlb measured is representative of the entire plasma volume.

Formulas (1)–(4) are used at all time points. When considering serial time points (n + 1 versus n) in the predetermined protocol, the HbM at time n + 1 can be derived from the value at time n, and the measured gain and loss of hemoglobin in that time interval is calculated as follows:

(5) $${HbM}_{n+1}={HbM}_{n}-bleeding\;volume\times mean\;SHb+hemoglobin\;transfusion$$

When MHb_n+1_ from Equation (5) is substituted into Equation (3), together with SHb_n+1_ and SHct_n+1_, PV_n+1_ can be estimated. This value can be inserted into Formula (4) together with SAlb_n+1_ to calculate IVAM_n+1_, representing the albumin mass at time point n + 1 related only to SHb, SHct, and the measured gain and loss of hemoglobin. IVAM’ is another method of direct evaluation of the albumin mass balance by considering the gains and losses of albumin over time. An apostrophe indicates that these IVAM’ values are calculated differently. Albumin gain was calculated using the albumin administration and albumin content in the transfusions. Albumin losses were calculated using the estimated bleeding loss in gauzes and suction containers or albumin measurement in Jackson–Pratt drains as follows:

(6) Albumin loss=bleeding volume×mean SAlb×(1−mean SHct)

The cumulative difference between these two estimations over time is shown in this article as the cumulative perioperative Alb_shift_, supposedly in the extracellular space:

(7) $$Cumulative\;perioperative\;Albshift={IVAM}^{\prime }-IVAM$$

Finally, the fractional plasma volume dilution at time n (fPVD_n_) was associated with the baseline plasma volume PV0 as follows:

(8) $${fPVD}_{n}=\frac{{PV}_{n}}{{PV}_{0}}$$

Similar fPVD estimations act as in-data in volume kinetic analysis [17].

The albumin shift per minute was calculated by first determining the total albumin shift over each specified time interval. These shifts were then converted into per-minute values by dividing the total albumin shift by the number of minutes within each interval. While the time intervals indeed span days, this method allowed the standardization of the rate of albumin change over time. We ensured that individual measurements were accurately recorded and appropriately averaged to reflect the continuous rate of albumin shift.

### 2.2. Data Collection

Data were extracted from electronic medical records and analyzed based on the presence or absence of major complications, which was the primary outcome in the current analysis. Major complications were defined as complications requiring surgical, endoscopic, or radiological intervention (Clavien–Dindo Class III) or life-threatening complications requiring ICU management (Clavien–Dindo Class IV) [18,19,20,21].

The preoperative data included various parameters such as age, sex, height, weight, comorbidities, Eastern Cooperative Oncology Group score, Performance Status Scale score, prior surgical score, and primary cancer. The Eastern Cooperative Oncology Group score was used to evaluate the performance status [22]. The Performance Status Scale score was used to estimate the extent of previous surgical intervention [23]. Intraoperative data included peritoneal cancer index, number of resected organs, liver resection, diaphragm injury, completeness of cytoreduction, crystalloid input, colloid input, packed red blood cell transfusion, fresh frozen plasma transfusion, albumin input, urine output, blood loss, norepinephrine use, need for vasopressin, operation time, and anesthesia time. The postoperative data included complications, mortality rates, requirement for vasopressors, and duration of ICU and hospital stay. Pulmonary disease was defined as any disorder affecting the pulmonary system, including pneumonia. Infection was defined as any inflammatory disease, excluding pneumonia. Ventilatory difficulties caused by diaphragm injury were not included as pulmonary disease.

### 2.3. Statistical Analyses

The reported incidence of major postoperative complications (Clavien–Dindo Class III–V) in patients undergoing CRS + HIPEC is 22.7% [19]. For this investigation, a minimum of 106 patients was deemed necessary to replicate the incidence of major complications, ensuring a two-sided 95% confidence interval (CI) width of 8%.

The data are presented as the median (interquartile range [IQR]), mean ± standard deviation, or n (%), as applicable. Patients were categorized based on the occurrence of major complications. Student’s t-test or Mann–Whitney U test was used for comparing continuous variables after the normality test. Chi-square or Fisher’s exact test (if the number in a cell was less than 5) was used for comparing categorical variables.

Univariate analysis was employed to examine the variables that exhibited significant differences between groups based on the presence of major complications. This regression incorporated albumin-related variables along with covariates comprising factors that showed statistical significance in the univariate analysis, using a stepwise method. Anesthetic time was excluded from the analysis because of multicollinearity with operation time. We employed three multivariate logistic regression models based on the selection of covariates. The covariates included operative time, crystalloid input, colloid input, blood loss, diaphragmatic injury, PCI score > 19, and number of anastomoses.

Receiver operating characteristic (ROC) curves were drawn for albumin-related variables that were statistically significant in the multivariate logistic regression analysis. The areas under the ROC curve (AUROCs) for albumin-related variables were compared to identify variables that demonstrated a stronger association. Statistical analyses were performed using SAS version 9.4 (SAS Institute, Cary, NC, USA) and R version 4.0.3 (The R Foundation for Statistical Computing, Vienna, Austria). Statistical significance was set at *p* < 0.05.

## 3. Results

**Figure 1:**
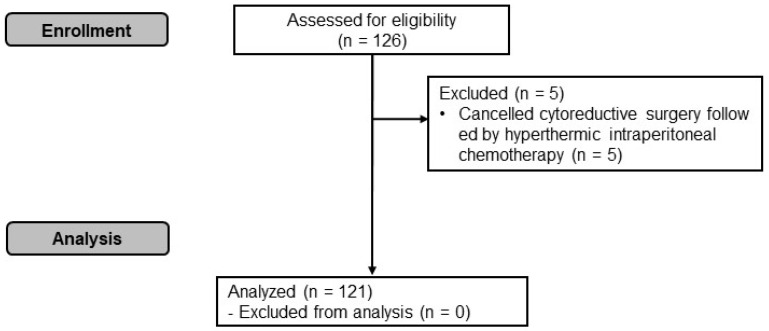
Flow chart of the participant recruitment process.

We enrolled 126 patients, of whom 5 were excluded because of the cancellation of CRS + HIPEC due to inoperable status (Figure 1).

In total, 121 patients were included in the final analysis. Major complications were observed in 25 (21%) patients (Table 1).

There were no statistically significant relationships between major complications and other demographic characteristics. The preoperative albumin level was comparable between patients who experienced major complications and those who did not. The PCI score had a *p*-value of 0.051, approaching statistical significance but not demonstrating a significant difference. Patients with a PCI score >19 were more represented among patients with major complications. The number of anastomoses was higher in patients with major complications. Univariable logistic regression revealed that the number of anastomoses and a PCI score > 19 were significantly associated with an increased risk of major complications (*p*-value of 0.012 and 0.006, respectively). The incidence of diaphragmatic injury was higher in patients with major complications. More patients without major complications had a score of 0 (no peritoneal nodule observed) for the completeness of cytoreduction. Intraoperative crystalloid input, colloid input, albumin input, blood loss, operation time, and anesthesia time were higher and urine output was lower in patients with major complications. Among these complications, postoperative pulmonary disease, gastrointestinal leakage, and infection were more prevalent in patients with major complications. The duration of ICU and hospital stays and mortality rate were comparable between the groups. Patients with major complications received significantly greater volumes of colloid infusion. Multivariable logistic regression showed that colloid infusion was not an independent risk factor for major complications, the primary endpoint of our study.

Among the minor complications, four patients with pulmonary disease required ventilatory support and ICU care due to acute respiratory distress syndrome resulting from pneumonia. Nine patients with anastomotic leakage underwent reoperation for reanastomosis. Three patients with ileus required surgery to relieve the obstruction. Six patients with infection needed vasopressors and ICU care due to septic shock. Among the minor complications, two patients with pulmonary disease were treated with antibiotics. For acute kidney injury, four patients were treated with fluid management and two with diuretics. Six patients with ileus received conservative management. Four patients with infection were treated with antibiotics.

**Figure 2:**
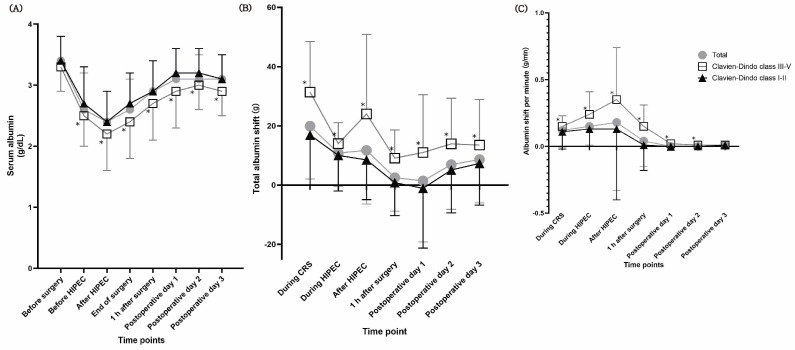
Albumin kinetics. (**A**) serum albumin level; (**B**) albumin shift; (**C**) albumin shift per minute. The serum albumin level was lower in patients with major complications (Clavien–Dindo class III–V) before HIPEC; after HIPEC; at the end of surgery; 1 h after surgery; and on postoperative days 1, 2, and 3 compared with that in patients without major complications. The total albumin shift was higher in patients with major complications at all time points. The albumin shift per minute was higher in patients with major complications during CRS; HIPEC; after HIPEC; at 1 h after surgery; and on postoperative days 1, 2, and 3 compared with that in patients without major complications. HIPEC, hyperthermic intraperitoneal chemotherapy; CRS, cytoreductive surgery. * *p* < 0.05 compared with patients without major complications.

SAlb levels decreased after surgery and subsequently increased (Figure 2 and Table S1). The SAlb concentration before CRS was comparable between patients with and without major complications. Patients with major complications exhibited lower SAlb levels on POD 1 and POD 3. ΔAlb during the 3 days after surgery and during surgery and on POD 3 was higher in patients with major complications than in patients without major complications. Alb_shift_ at all time points was higher in patients with major complications. A comparison of perioperative albumin loss according to the occurrence of each complication is presented in Table S2. Patients with anastomotic leakage showed a significantly higher ΔAlb during surgery and on POD 3 and Alb_shift_ at all time points compared with the scores of those without anastomotic leakage. Alb_shift_ during the 3 days after surgery and during surgery and on POD 3 was higher in patients with infection. Patients with acute kidney injury showed a significantly higher Alb_shift_ during surgery. Patients with pulmonary complications demonstrated a significantly higher Alb_shift_ during the 3 days after surgery. Patients who were readmitted showed a significantly higher Alb_shift_ at all time points.

Multivariate logistic regression analysis revealed that the SAlb concentration at all time points was not an independent risk factor for major complications (Table 2). Multivariable logistic regression analysis was conducted, considering variables related to albumin as the main independent variables (Main IV). For each main independent variable, the following covariates were included in the model for adjustment: duration of surgery, intraoperative crystalloid administration, intraoperative colloid administration, intraoperative blood loss, and intraoperative diaphragmatic damage.

ΔAlb observed both during surgery and on POD 3 was not identified as an independent risk factor for major complications. Alb_shift_ during surgery (*p* < 0.001, odds ratio: 1.039 [1.018–1.060]), during the 3 days after surgery (*p* < 0.001, odds ratio: 1.058 [1.031–1.087]), and between surgery and POD 3 (*p* < 0.001, odds ratio: 1.045 [1.026–1.065]) was identified as an independent risk factor for major complications. Alb_shift_ before HIPEC (*p* = 0.023, odds ratio: 1.040 [1.006–1.076) and after HIPEC (*p* = 0.005, odds ratio: 1.045 [1.013–1.078) was also identified as an independent risk factor for major complications.

**Figure 3:**
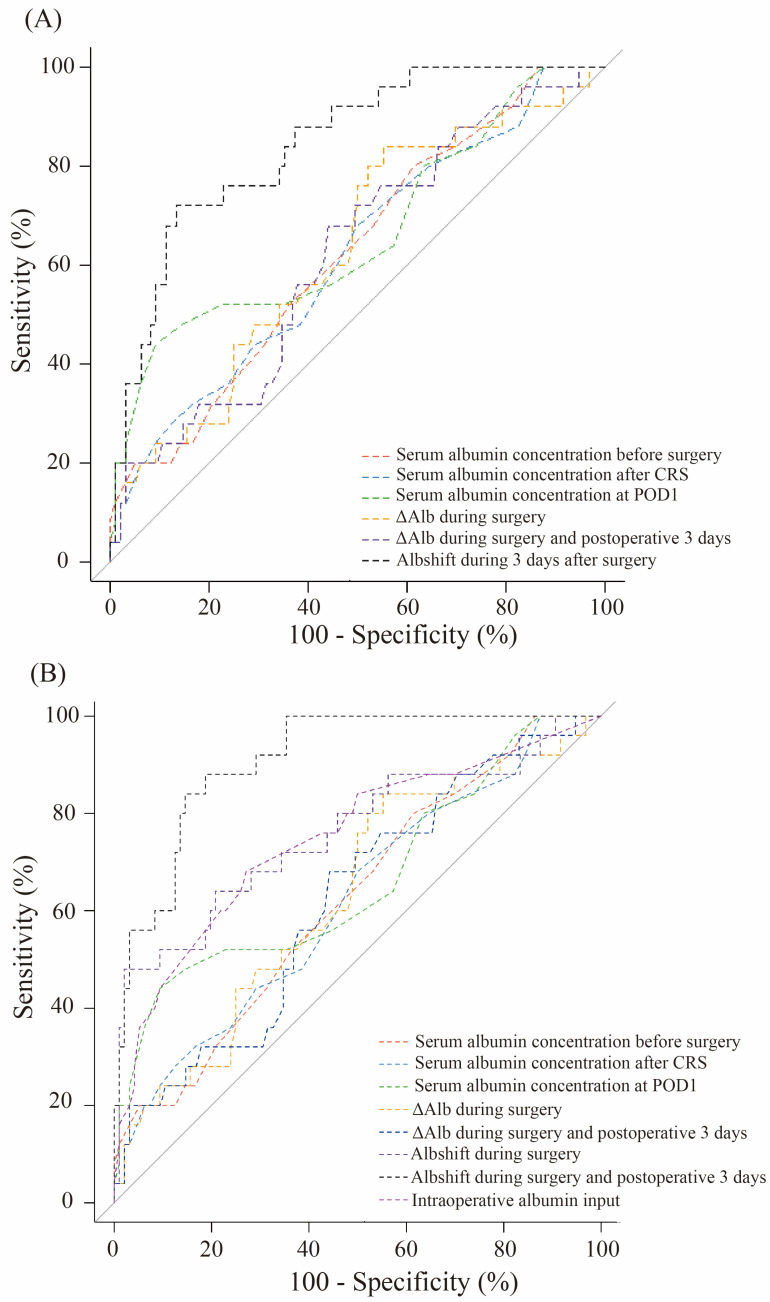
Receiver operating characteristic curves to detect stronger association with major complications. (**A**) Comparison of albumin shift during 3 days after surgery; (**B**) Comparison of albumin shift during surgery and on postoperative day 3. Major complications were defined as a Clavien–Dindo Class III–IV in 30 days. The ∆Alb level was defined as the change in the serum albumin level over time. Alb_shift_ was defined as the pattern of changes in serum albumin levels over time and the calculated albumin extravasation, based on previous studies. CRS, cytoreductive surgery; POD, postoperative day; ∆Alb, albumin decrease; HIPEC, hyperthermic intraperitoneal chemotherapy; Alb_shift_, albumin shift.

The ROC analysis of statistically significant albumin variables revealed that the AUROC values of Alb_shift_ during the 3 days after surgery and Alb_shift_ during surgery and on POD 3 were 0.843 (95% CI: 0.761–0.924) and 0.910 (95% CI: 0.856–0.964), respectively (Figure 3 and Table S3). The comparative analysis of AUROC revealed that the AUROC of Alb_shift_ during the 3 days after surgery and during surgery and on POD 3 significantly exceeded those of other albumin-related variables.

## 4. Discussion

The present study assessed perioperative ∆Alb and Alb_shift_ in patients undergoing CRS + HIPEC and their associations with major postoperative complications. Alb_shift_ during 3 days after surgery and Alb_shift_ during surgery and on POD 3 were more strongly associated with complications than were other albumin-related variables.

Moreover, the precise measurement of albumin shifts immediately after the cytoreductive phase and subsequent measurement after HIPEC have provided valuable insights. These distinctions help to clarify the contribution of each surgical phase to albumin loss and its association with postoperative complications, as now reflected in our findings.

In this investigation, the incidence of major postoperative complications was 21%, aligning with findings from a previous investigation [24]. Major postoperative complications (Clavien–Dindo Class III–V) were associated with diminished survival rates in patients undergoing CRS + HIPEC [25]. Major complications also increased the duration of hospitalizations and economic costs [26]. Although we did not conduct a cost–benefit analysis, it is important to note that the occurrence of major complications necessitating procedural intervention and/or ICU admission can significantly increase medical costs. Additionally, these complications can prolong hospitalization, further escalating the economic burden. Diverse protocols encompassing fluid management, hemodynamic monitoring, early nutritional support, and multimodal analgesia have been proposed to reduce complications in patients undergoing CRS + HIPEC [27]. Identifying risk factors for major postoperative complications in this cohort may aid in prognostication and devising treatment strategies. The perioperative variables other than albumin-related variables were comparable between patients with and without major postoperative complications. Albumin-related variables such as the SAlb concentration after CRS and on POD 1 and POD 3, ΔAlb during surgery and during surgery and on POD 3, and Alb_shift_ at all time points exhibited notable differences between patients with and without Clavien–Dindo Class III–V complications. This study demonstrates the strong association between albumin reduction during major surgery and postoperative morbidity in patients undergoing CRS + HIPEC.

In this investigation, the median Alb_shift_ during surgery and POD 3 was 127.5 g (IQR: 71.9) in patients with complications, notably higher than that in a previous investigation [2]. The mechanisms behind this albumin shift in major surgeries primarily point to capillary leakage [2]. Overhydration and inflammation are related to capillary leakage. CRS + HIPEC is an extensive procedure including a massive surgical maneuver and chemotherapy. The procedure’s complexity can lead to substantial bleeding, which was higher in this investigation than in previous studies [2]. This increased blood loss potentially necessitated increased fluid replacement. The inevitable inflammatory response triggered by surgical manipulation and intraperitoneal chemotherapy contributes to heightened levels of inflammatory markers such as C-reactive protein and pancreatic stone protein in patients undergoing CRS + HIPEC [28]. These factors associated with overhydration and inflammation may contribute to the increased capillary leakage and relatively higher Alb_shift_ in our investigation.

Variables related to decreased albumin levels were associated with major complications (Clavien–Dindo Class III–V) in our study. A postoperative decrease in SAlb is related to increased complications after major abdominal surgeries [29,30,31]. The relationship between albumin-related variables and complications in this investigation aligns with that reported in previous investigations. SAlb plays an important role in cell growth stabilization, DNA replication, hormone homeostasis maintenance, and systemic inflammation regulation [32]. These critical functions of SAlb may account for the robust correlation observed between albumin levels and major postoperative complications. We additionally assessed ΔAlb and Alb_shift_, which indicate relative albumin changes and the rate of albumin changes. This investigation demonstrated that Alb_shift_ during the first 3 days after surgery and Alb_shift_ during surgery and on POD 3 are more strongly associated with postoperative complications than are other albumin-related variables. These Alb_shift_ variables should be evaluated in future investigations and can be used to predict complications in clinical practice. As Alb_shift_ indicates albumin loss by capillary leakage [2], meticulous fluid replacement may be advisable in patients exhibiting significant Alb_shift_, because fluid replacement from capillary leakage can induce interstitial edema and exacerbate capillary leakage. Considering albumin’s role in stabilizing the glycocalyx [33] and impeding heparin-binding protein, which is known to influence endothelial patency [34], albumin replacement may be recommended for this patient population. The albumin replacement protocol, however, is still under investigation [35]. Based on ongoing research and future studies, albumin replacement may be considered in patients undergoing CRS + HIPEC. Postoperative tissue edema in patients undergoing abdominal surgery could be associated with increased complications [36]. Monitoring albumin levels from multiple perspectives can be clinically advantageous, as correcting albumin deficiencies with albumin administration may help to prevent edema [37]. Furthermore, perioperative nutritional support is a critical component of Enhanced Recovery After Surgery protocols and is essential for achieving better surgical outcomes [38]. Monitoring albumin levels can provide a benchmark for assessing whether perioperative nutrition is adequate and appropriately managed [39].

Diaphragm injury is recognized as an independent factor associated with poor prognosis in patients undergoing HIPEC and CRS [40]. In our investigation, we observed differing incidences of diaphragm injury between patients with major complications and those without. Specifically, patients who experienced major complications had a higher incidence of diaphragmatic injury. To account for this, we included the incidence of diaphragm injury as a covariate in our logistic regression analysis. This adjustment was made to minimize the potential confounding effect of diaphragm injury on the albumin variables, ensuring that our assessment of albumin shifts and their association with complications remains accurate. Anastomotic leakage following the CRS + HIPEC procedure is associated with significant mortality [41], and the number of anastomoses is a recognized risk factor for this complication. We compared the number of anastomoses between groups to identify potential disparities and, given the observed differences, this variable was included as a covariate in the logistic regression analysis. Operating time and crystalloid input levels were also included as covariates due to their potential association with increased bowel edema and anastomotic leakage [42,43]. Additionally, both operating time and ventilation duration may be linked to a higher incidence of pulmonary complications [44,45]. In our study, patients who experienced major complications had longer operating times and a higher incidence of pulmonary complications, which were included in our definition of major complications. However, due to the limited number of cases, a separate analysis focusing solely on pulmonary complications was not feasible. Given the critical nature of these complications, future research should specifically investigate this aspect in patients undergoing CRS followed by HIPEC. In the multivariate analysis, the primary focus was on albumin-related variables. Although operating time and blood loss were included as covariates, they were not examined as independent risk factors for major complications. However, we acknowledge that these parameters have been consistently identified as significant predictors of postoperative morbidity in previous studies [24,41,42,43,45]. Parameters such as operating time, blood loss, blood transfusion, infusion volume, and the number and type of surgical procedures are well-established surrogate markers for the complexity of surgery and the risk of severe postoperative complications. Regarding Alb_shift_, our analysis indicates that it is not associated with specific postoperative complications. Consequently, the utility of determining albumin shift intraoperatively and postoperatively may be limited unless these measurements guide therapeutic interventions, such as albumin substitution during or after surgery.

The present study had some limitations. First, the study was conducted retrospectively, highlighting the need for future prospective studies to comprehensively assess the efficacy of aggressive albumin replacement in patients exhibiting severe ΔAlb or Alb_shift_ during CRS + HIPEC. CRS + HIPEC is an extensive procedure involving a wide range of interventions. Despite the observed relationship between preoperative albumin deficiency and high morbidity and low survival rates in patients undergoing CRS + HIPEC [11,12], the specific amount of albumin shift during this particular surgery, which could have more significant implications, had never been assessed. Hence, we investigated this metric during surgery and the acute postoperative phase. However, we acknowledge that there might be various factors contributing to major complications. To address this, we performed a multivariate analysis to encompass these factors. Second, calculating Alb_shift_ is difficult because of the use of intricate mathematical formulae. However, our study successfully utilized a preset computerized formula to determine Alb_shift_, simplifying its assessment. Third, all participants in this investigation underwent CRS + HIPEC, a procedure associated with notably high morbidities. Consequently, the generalizability of our findings to other surgical patient cohorts is limited. Fourth, our investigation did not show differences in mortality with respect to albumin levels. Further investigations with enhanced statistical power may be necessary to ascertain the impact of albumin decrease on the outcomes in this population. Fifth, the PCI index determines the extent of peritoneal disease and partly the extent of cytoreduction and all resections. In this investigation, however, the PCI index was comparable between patients who experienced major complications and those who did not. Specifically, pseudomyxoma peritonei and mesothelioma with high PCI scores, which are associated significant capillary leaks and disseminated coagulation disorders, were not identified as independent risk factors. This result may be due to the small number of cases with high PCI scores. Larger-scale studies might include these factors as potential risk indicators. Further research regarding the PCI index, albumin-related variables, and patient outcomes is necessary. Sixth, we calculated the albumin shift per minute by converting albumin changes measured during each phase into a per-minute rate. This approach, while standardizing the rate of change, may introduce challenges in accurately interpreting the continuous nature of albumin dynamics, which could affect the precision of our findings when the data points are spread over several days. Seventh, our investigation did not explicitly differentiate between the effects of substituted (exogenous) albumin and endogenous albumin. It is important to note that while substituted albumin is administered for therapeutic use, its comparison with naturally occurring endogenous albumin is inherently limited. Eighth, our study was conducted retrospectively, which presents several limitations, including selection bias, confounding variables, and challenges in performing multivariate analyses. To overcome these limitations, a prospective study that measures albumin shifts, involves aggressive albumin replacement, and assesses the impact on postoperative complications and mortality is necessary. Such an approach would enable a more comprehensive and controlled evaluation of albumin dynamics and their clinical implications, thereby providing more accurate and reliable data on the effects of albumin replacement strategies in the context of CRS + HIPEC.

## 5. Conclusions

In conclusion, serum ΔAlb and Alb_shift_ were observed perioperatively in patients undergoing CRS + HIPEC, and changes in albumin levels were associated with major postoperative complications. Alb_shift_ was more strongly associated with serious complications than were other albumin-related variables. We recommend the use of Alb_shift_ in the clinical management of patients undergoing CRS + HIPEC.

## Figures and Tables

**Table 1:** Demographic and perioperative data according to the occurrence of major complications.

	Clavien–Dindo Class III–V		*p*-Value
	Yes (n = 25)	No (n = 96)	
Preoperative data			
Age, years	55 (14)	56 (18)	0.758
Female (%)	13 (52%)	49 (51%)	0.932
Male (%)	12 (48%)	47 (49%)	
Height, cm	164 ± 8	163 ± 8	0.308
Weight, kg	59.4 (13.0)	59.9 (12.6)	0.347
Albumin, g/dL	3.5 (0.6)	3.4 (0.3)	0.757
Comorbidity (%)			
Hypertension, yes	9 (36%)	19 (20%)	0.087
Diabetes, yes	4 (16%)	13 (14%)	0.753
Cerebrovascular disease, yes	1 (4%)	3 (3%)	0.827
Kidney disease, yes	0 (0%)	1 (1%)	1.000
ECOG score (0/1/2/3/4/5, %)	20 (80%)/3 (12%)/2 (8%)/0 (0%)/0 (0%)/0 (0%)	78 (81%)/10 (10%)/5 (5%)/3 (3%)/0 (0%)/0 (0%)	0.777
PSS (0/1/2/3, %)	11 (44%)/10 (40%)/3 (12%)/1 (4%)	33 (34%)/32 (33%)/18 (19%)/13 (14%)	0.417
Primary cancer (%)			0.312
Colorectal cancer, yes	20 (80%)	89 (93%)	
Gastric cancer, yes	2 (8%)	9 (9%)	
Pseudomyxoma peritonei, yes	1 (4%)	5 (5%)	
Mesothelioma, yes	1 (4%)	3(3%)	
Intraoperative data			
PCI score	21 (23)	13 (21)	0.051
PCI score > 19, yes	15 (60%)	28 (29%)	0.009
Mesothelioma or pseudomyxoma peritonei with a PCI score greater than 19, yes	2 (8%)	5 (5%)	0.633
Lymph node dissection, yes	8 (32%)	30 (31%)	1.000
Vascular resection, yes	1 (4%)	4 (4%)	1.000
Number of anastomosis	1.9 ± 1.0	1.3 ± 1.0	0.009
Number of resected organs	5.6 ± 2.6	4.7 ± 2.6	0.102
Resected organs			
Colon, yes	17 (68%)	67 (70%)	0.863
Intestine, yes	16 (64%)	41 (43%)	0.057
Stomach, yes	3 (12%)	11 (12%)	1.000
Pancreas, yes	0 (0%)	2 (2%)	1.000
Gallbladder, yes	4 (16%)	30 (31%)	0.210
Liver, yes	7 (28%)	18 (19%)	0.309
Diaphragm, yes	15 (60%)	28 (29%)	0.004
Uterus, yes	6 (24%)	17 (18%)	0.475
Ovary, yes	6 (24%)	25 (26%)	0.835
Omentum, yes	12 (48%)	58 (60%)	0.263
Peritoneum, yes	20 (80%)	69 (72%)	0.412
Spleen, yes	7 (28%)	11 (12%)	0.056
Bladder, yes	8 (32%)	18 (19%)	0.175
Diaphragmatic injury, yes	15 (60%)	28 (29%)	0.004
Completeness of cytoreduction (0/1/2/3, %)	12 (48%)/6 (24%)/3 (12%)/4 (16%)	68 (71%)/5 (5%)/10 (10%)/13 (14%)	0.024
Crystalloid input, mL	7050 (3675)	5400 (2538)	0.022
Colloid input, mL	760 ± 542	467 ± 462	0.018
pRBC input, unit	2.0 ± 4.6	1.2 ± 2.2	0.395
FFP input, unit	1.0 ± 3.1	0.3 ± 1.2	0.305
Albumin input, mL	60 (60)	23 (50)	<0.001
Urine output, mL	1650 (1008)	1000 (889)	0.007
Blood loss, mL	1891 ± 2187	1047 ± 951	0.070
Norepinephrine, mcg	1558 ± 3202	1017 ± 1753	0.466
Need of vasopressin, yes	2 (8%)	15 (16%)	0.520
Operation time, min	710 (410)	443 (304)	0.011
Anesthesia time, min	795 (403)	548 (308)	0.013
Postoperative data			
ICU duration, day	1.8 ± 2.1	1.1 ± 1.1	0.146
Complications			
Pulmonary disease, yes	4 (16%)	2 (2%)	0.004
Acute kidney injury, yes	0 (0%)	6 (6%)	0.343
Gastrointestinal anastomosis leakage, yes	9 (36%)	0 (0%)	<0.001
Ileus, yes	3 (12%)	6 (6%)	0.329
Infection, yes	6 (24%)	4 (4%)	0.005
Mortality, yes	7 (28%)	15 (16%)	0.153
Need of vasopressor, yes	6 (24%)	18 (19%)	0.558
Length of hospital stay, day	18 (23)	15 (6)	0.098

ECOG, Eastern Cooperative Oncology Group Performance Status Scale; PSS, prior surgical score; PCI, peritoneal cancer index; pRBC, packed red blood cell; FFP, fresh frozen plasma; ICU, intensive care unit. Among the minor complications, four patients with pulmonary disease required ventilatory support and ICU care due to acute respiratory distress syndrome resulting from pneumonia. Nine patients with anastomotic leakage underwent reoperation for reanastomosis. Three patients with ileus required surgery to relieve the obstruction. Six patients with infection needed vasopressors and ICU care due to septic shock. Among the minor complications, two patients with pulmonary disease were treated with antibiotics. For acute kidney injury, four patients were treated with fluid management and two with diuretics. Six patients with ileus received conservative management. Four patients with infection were treated with antibiotics. Data are presented as the median (interquartile range), number (%), or mean ± standard deviation.

**Table 2:** Multivariate logistic regression models of the association between albumin-related variables and major complications.

	Main IV	Covariates						
Main IV		Operation Time	Crystalloid Input	Colloid Input	Blood Loss	Number of Anastomoses	Diaphragmatic Injury (Yes)	PCI Score > 19
∆Alb during surgery								
Odds ratio (95% CI)	1.023 (0.994–1.053)	1.002 (0.998–1.006)	1.000 (1.000–1.000)	1.000 (0.999–1.001)	1.000 (1.000–1.001)	1.456 (0.833–2.542)	1.498 (0.449–5.004)	2.511 (0.898–7.023)
*p*-value	0.128	0.346	0.239	0.890	0.279	0.187	0.511	0.079
∆Alb during surgery and POD 3								
Odds ratio (95% CI)	1.052 (0.998–1.108)	1.001 (0.997–1.005)	1.000 (0.999–1.000)	1.000 (0.999–1.001)	1.000 (1.000–1.001)	1.622 (0.909–2.896)	1.546 (0.462–5.169)	2.955 (1.02–8.532)
*p*-value	0.060	0.681	0.327	0.854	0.197	0.102	0.480	0.045
Alb_shift_ during surgery								
Odds ratio (95% CI)	1.037 (1.016–1.058)	1.002 (0.998–1.006)	1.000 (0.999–1.000)	1.000 (0.999–1.001)	1.000 (1.000–1.001)	1.374 (0.748–2.525)	0.948 (0.244–3.686)	2.523 (0.803–7.930)
*p*-value	0.001	0.365	0.208	0.957	0.235	0.306	0.939	0.113
Alb_shift_ before HIPEC								
Odds ratio (95% CI)	1.040 (1.006–1.076)	1.040 (1.006–1.076)	1.040 (1.006–1.076)	1.040 (1.006–1.076)	1.040 (1.006–1.076)	1.040 (1.006–1.076)	1.040 (1.006–1.076)	1.040 (1.006–1.076)
*p*-value	0.023	0.492	0.320	0.700	0.363	0.177	0.811	0.102
Alb_shift_ after HIPEC								
Odds ratio (95% CI)	1.045 (1.013–1.078)	1.045 (1.013–1.078)	1.000 (0.999–1.000)	1.000 (0.999–1.001)	1.000 (0.999–1.001)	1.334 (0.750–2.373)	1.305 (0.357–4.770)	2.845 (0.948–8.538)
*p*-value	0.005	0.340	0.198	0.945	0.217	0.327	0.687	0.062
Alb_shift_ during 3 days after surgery								
Odds ratio (95% CI)	1.058 (1.029–1.088)	1.000 (0.996–1.005)	1.000 (0.999–1.000)	1.001 (0.999–1.002)	1.000 (1.000–1.001)	1.420 (0.726–2.778)	4.186 (0.977–17.939)	0.886 (0.232–3.387)
*p*-value	<0.001	0.942	0.291	0.499	0.292	0.306	0.054	0.859
Alb_shift_ during surgery and POD 3								
Odds ratio (95% CI)	1.046 (1.025–1.067)	1.001 (0.996–1.006)	1.000 (0.999–1.000)	1.000 (0.999–1.002)	1.000 (1.000–1.001)	1.444 (0.663–3.143)	1.912 (0.415–8.822)	0.917 (0.214–3.924)
*p*-value	<0.001	0.703	0.169	0.748	0.213	0.355	0.406	0.907

IV, independent variable; PCI, peritoneal cancer index; POD, postoperative day; ∆Alb, albumin decrease; Alb_shift_, albumin shift; HIPEC, hyperthermic intraperitoneal chemotherapy; major complications were defined as a Clavien–Dindo Class III–IV in 30 days. The ∆Alb level was defined as the change in the serum albumin level over time. Alb_shift_ was defined as the pattern of changes in serum albumin levels over time and calculated albumin extravasation, based on previous studies. Multivariable logistic regression analysis was conducted, considering variables related to albumin as the main independent variables (Main IV). For each main independent variable, the following covariates were included in the model for adjustment: duration of surgery, intraoperative crystalloid administration, intraoperative colloid administration, intraoperative blood loss, and intraoperative diaphragmatic damage.

## Data Availability

The data of this investigation are shared in Mendeley Data. Kim, Hyun-Chang (2024), “Leakage of albumin levels during cytoreductive surgery and hyperthermic intraperitoneal chemotherapy predicts the major complications”, Mendeley Data, V2, doi: 10.17632/bwvtk53mvj.2.

## Supplementary Materials

The following supporting information can be downloaded at: https://www.mdpi.com/article/10.3390/cancers16162874/s1, Table S1: Perioperative albumin-related parameters according to major complications; Table S2: Complications and albumin-related variables; Table S3: Comparison of area under the receiver operating characteristic curve for albumin-related variables.
