# Supplementary material for: Albumin Leakage Level during Cytoreductive Surgery and Hyperthermic Intraperitoneal Chemotherapy Is Associated with Major Complications
Source: Cancers (Basel). 2024 Aug 19;16(16):2874. doi: 10.3390/cancers16162874 (PMC11352347; doi:10.3390/cancers16162874)
Supplement: Supplementary file 1 [file cancers-16-02874-s001.zip › cancers-3139050-supplementary.pdf]

Supplementary Materials

Table S1. Perioperative albumin-related parameters according to major complications.

Albumin-related variables		Clavien–Dindo Class III–V		
	Total	Yes (n = 25)	No (n = 96)	*P* value
Serum albumin				
Concentration, g/dL				
Before surgery	3.4 ± 0.4	3.3 ± 0.4	3.4 ± 0.4	0.073
After CRS (before HIPEC)	2.6 ± 0.5	2.4 ± 0.6	2.7 ± 0.5	0.046
POD1	3.1 ± 0.5	2.9 ± 0.6	3.2 ± 0.4	0.005
POD2	3.1 ± 0.4	3.0 ± 0.4	3.2 ± 0.4	0.071
POD3	3.1 ± 0.4	2.9 ± 0.4	3.1 ± 0.4	0.038
ΔAlb, %				
During surgery	21 ± 22	28 ± 19	19 ± 22	0.034
During surgery and 3 days postoperatively	7 ± 14	12 ± 12	6 ± 14	0.032
Alb_{shift}, g				
During surgery	36.7 (32.2)	63.1 (68.8)	32.1 (26.6)	<0.001
During 3 days after surgery	18.4 (38.8)	44.5 (35.3)	14.7 (36.0)	<0.001
During surgery and 3 days postoperatively	57.4 (50.6)	127.5 (71.9)	48.5 (44.9)	<0.001

CRS, cytoreductive surgery; HIPEC, hyperthermic intraperitoneal chemotherapy; ΔAlb, albumin decrease; Alb_{shift}, albumin shift. ΔAlb was defined as the change in the serum albumin level over time. Alb_{shift} was defined as the pattern of changes in serum albumin levels over time and calculated albumin extravasation, based on previous studies. Data are presented as mean ± standard deviation or median (interquartile range).

Table S2. Complications and albumin-related variables.

Albumin-related variables	Anastomotic leakage		Infection		AKI		Pulmonary complication		Readmission		Mortality	
	Yes (n=9)	No (n=112)	Yes (n=10)	No (n=111)	Yes (n=6)	No (n=115)	Yes (n=6)	No (n=115)	Yes (n=21)	No (n=100)	Yes (n=22)	No (n=99)
Serum albumin concentration, g/dL												
Before CRS	3.5±0.4	3.4±0.4	3.3±0.5	3.4±0.4	3.4±0.5	3.4±0.4	3.1±0.6	3.4±0.4	3.3±0.4	3.4±0.4	3.4±0.5	3.4±0.4
After CRS (before HIPEC)	2.7±0.5	2.6±0.5	2.6±0.6	2.6±0.5	2.8±0.1	2.6±0.5	2.2±0.4	2.7±0.5	2.5±0.6	2.7±0.5	2.6±0.5	2.6±0.5
POD1	2.9±0.5	3.2±0.5	2.8±0.5	3.2±0.5	3.2±0.3	3.1±0.5	2.7±0.7	3.2±0.5	3.0±0.4	3.2±0.5	3.0±0.5	3.2±0.5
POD2	2.9±0.4	3.1±0.4	3.0±0.3	3.1±0.4	3.1±0.3	3.1±0.4	3.1±0.5	3.1±0.4	3.1±0.4	3.1±0.4	3.1±0.3	3.1±0.4
POD3	2.9±0.4	3.1±0.4	2.9±0.4	3.1±0.4	3.1±0.3	3.1±0.3	2.8±0.5	3.1±0.4	3.0±0.5	3.1±0.4	3.0±0.4	3.1±0.4
ΔAlb, %												
During surgery	23±10	20±23	23±15	20±23	13±15	21±22	31±11	20±22	23±24	20±22	20±15	21±23
During surgery 3 days postoperatively	18±13*	6±14	11±12	7±14	3±18	8±14	9±9	7±14	10±14	7±14	11±13	6±14
Alb_{shift}, g												
During surgery	101.1 (59.6)*	34.0 (28.8)	38.4 (66.1)	35.7 (32.1)	13.2 (24.9)*	38.5 (32.3)	35.4 (32.6)	36.9 (32.4)	53.8 (53.2)*	34.0 (28.3)	39.5 (43.0)	35.7 (29.6)
During 3 days after surgery	29.5 (51.5)*	17.6 (38.9)	39.6 (38.4)*	17.5 (37.8)	29.1 (22.8)	17.7 (39.8)	47.9 (16.8)*	17.5 (37.3)	44.5 (32.9)*	15.1 (35.5)	16.8 (37.7)	18.4 (38.7)
During surgery and 3 days postoperatively	139.2 (64.5)*	55.1 (46.0)	75.5 (98.9)*	55.6 (51.6)	35.2 (36.2)	58.0 (51.3)	80.4 (44.8)	56.2 (51.6)	89.7 (83.3)*	50.8 (47.1)	62.6 (35.7)	55.6 (52.1)

AKI, acute kidney injury; CRS, cytoreductive surgery; HIPEC, hyperthermic intraperitoneal chemotherapy; POD, postoperative day; ΔAlb, albumin decrease; Alb_{shift}, albumin shift. ΔAlb was defined as the change in the serum albumin level over time. Alb_{shift} was defined as the pattern of changes in serum albumin levels and calculated albumin extravasation, based on previous studies.

Data are presented as mean ± standard deviation or median (interquartile range). *$P < 0.05$.

Table S3. Comparison of area under the receiver operating characteristic curve for albumin-related variables.

	Area under the curve (95% confidence interval)	Comparison	*P* value
Serum albumin concentration before CRS	0.624 (0.504–0.745)		
Serum albumin concentration after CRS (before HIPEC)	0.620 (0.495–0.744)		
Serum albumin concentration on POD 1	0.664 (0.532–0.797)		
ΔAlb during surgery	0.630 (0.507–0.753)		
ΔAlb during surgery and 3 days postoperatively	0.621 (0.498–0.743)		
Alb_{shift} during surgery	0.758 (0.635–0.880)		
Alb_{shift} during 3 days after surgery	0.843 (0.761–0.924)		
		Serum albumin concentration before CRS	0.002
		Serum albumin concentration after CRS (before HIPEC)	0.006
		Serum albumin concentration on POD1	0.020
		ΔAlb during surgery	0.011
		Δ Alb during surgery and 3 days postoperatively	0.006
Alb_{shift} during surgery and 3 days postoperatively	0.910 (0.856–0.964)		
		Serum albumin concentration before CRS	<0.001
		Serum albumin concentration after CRS (before HIPEC)	<0.001
		Serum albumin concentration on POD1	0.001
		ΔAlb during surgery	<0.001
		ΔAlb during surgery and 3 days postoperatively	<0.001
		Alb_{shift} during surgery	0.004
		Alb_{shift} during 3 days after surgery	0.001
Intraoperative albumin input	0.749 (0.632–0.866)		

POD, postoperative day; ΔAlb, albumin decrease; CRS, cytoreductive surgery; HIPEC, hyperthermic intraperitoneal chemotherapy; Alb_{shift}, albumin shift. ΔAlb was defined as the change in the serum albumin level over time. Alb_{shift} was defined as the pattern of changes in serum albumin levels and calculated albumin extravasation, based on previous studies.
